# Supplementary material for: Irinotecan Induces Disease Remission in Xenograft Mouse Models of Pediatric MLL-Rearranged Acute Lymphoblastic Leukemia
Source: Biomedicines. 2021 Jun 23;9(7):711. doi: 10.3390/biomedicines9070711 (PMC8301450; doi:10.3390/biomedicines9070711)
Supplement: Supplementary file 1 [file biomedicines-09-00711-s001.zip › biomedicines-1255155-supplementary.pdf]

Supplemental Figure 1.

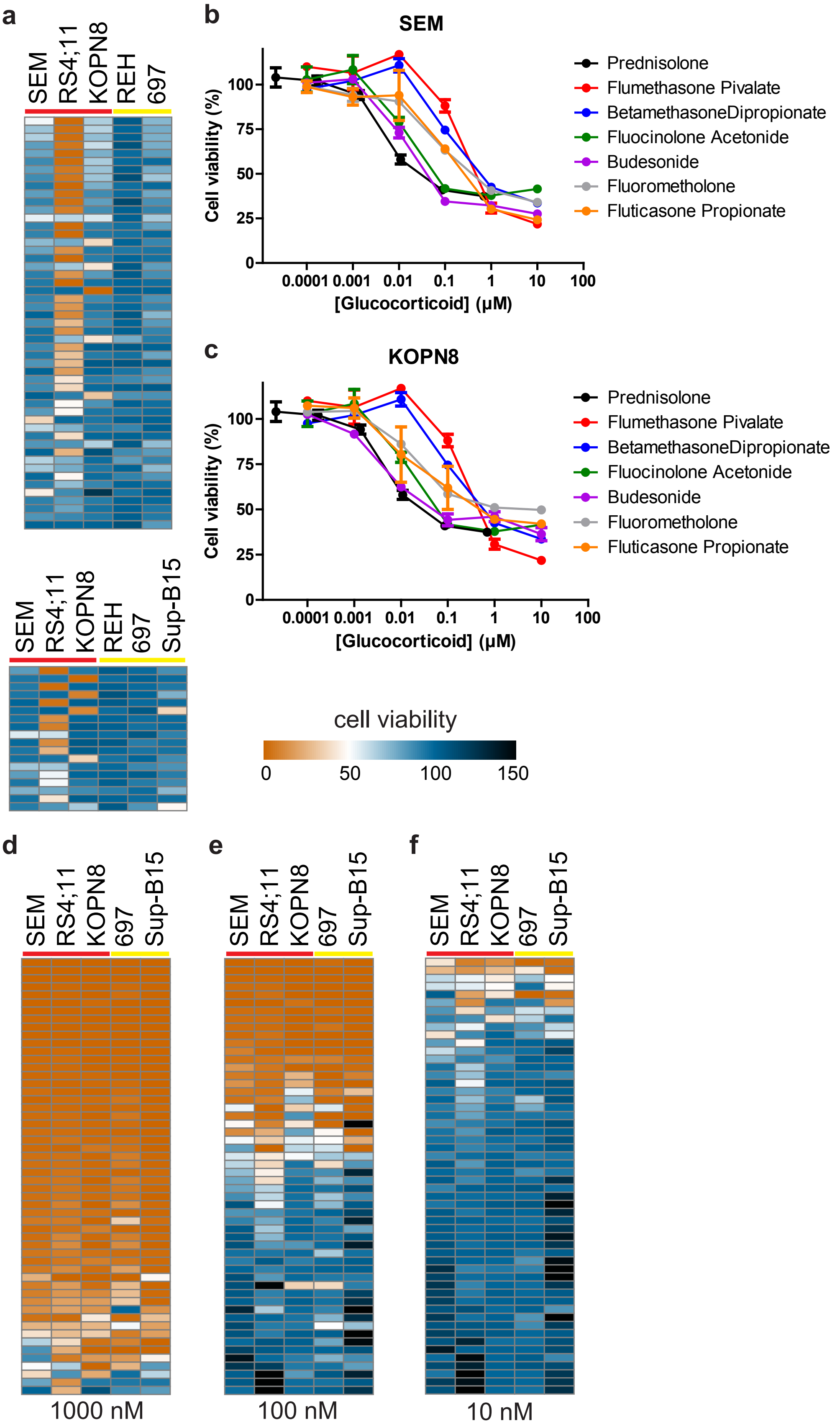

Supplemental Figure 1. (a) Heat map presentation of the screen data from 52 and 18 non-overlapping drugs (specifically affecting the *MLL*-rearranged cell lines) from the Spectrum library (top) and the Prestwick library (bottom), respectively. (b,c) Dose-response curves of a panel of corticosteroid drugs assessed cell viability against SEM (b) and KOPN8 (c). Shown are prednisolone (black), flumethasone (red), betamethasone (blue), fluocinolone (green), budesonide (purple), fluorometholone (grey) and fluticasone (orange). (d-f) Viability of the screen cell lines was assessed after exposure to 54 selected Spectrum/Prestwick library hits at 1 μM, 100 nM and 10 nM concentration, respectively.

Supplemental Figure 2.

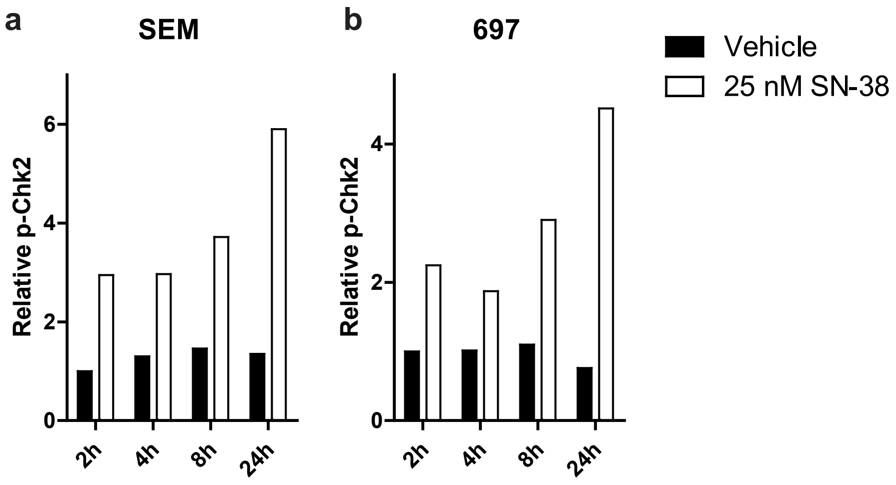

Supplemental Figure 2. (a,b) Quantification of the phosphorylated Chk2 levels relative to total Chk2 in vehicle or 25 nM SN-38 (black and white bars, respectively) exposed SEM and 697 cell lysates, respectively.

Supplemental Figure 3.

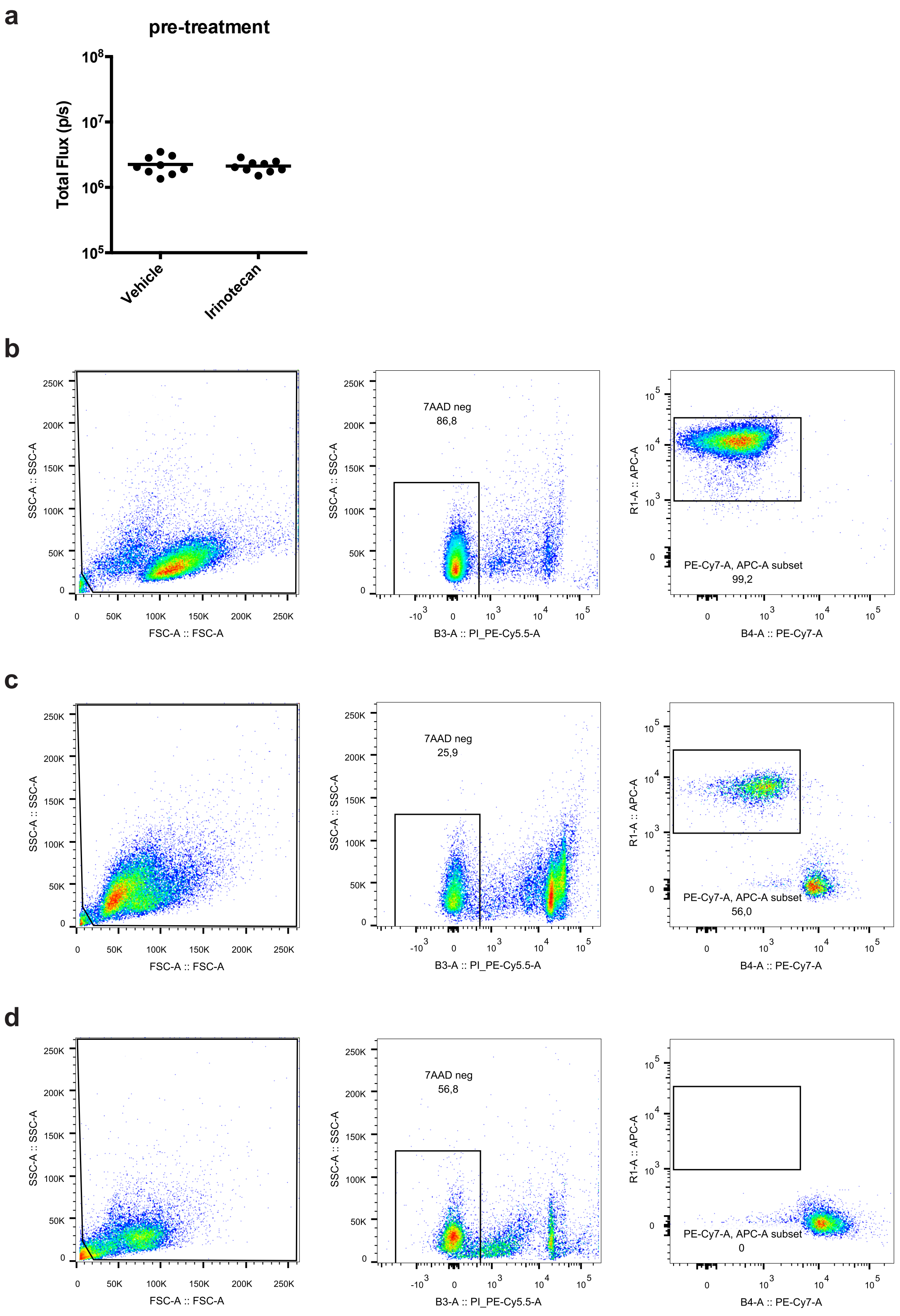

Supplemental Figure 3. (a) Quantification of the pre-treatment intra-vital imaging of the mice divided according to treatment group (vehicle or irinotecan) shows equal total flux. (b-d) Example gating for flow cytometry detection of leukemic cells in (homogenized) mouse tissues. Shown are gating of cellular events (removing debris; left), gating of live cells (7-AAD negative; center) and detection of human CD19 positive (APC channel) and mouse Cd45/mouse Ter119 negative (PE-Cy7 channel) cells (right) of pure SEM-SLIEW cells (b), and homogenized spleen samples of a vehicle treated mouse (c) and an irinotecan treated mouse (d).

Supplemental Figure 4.

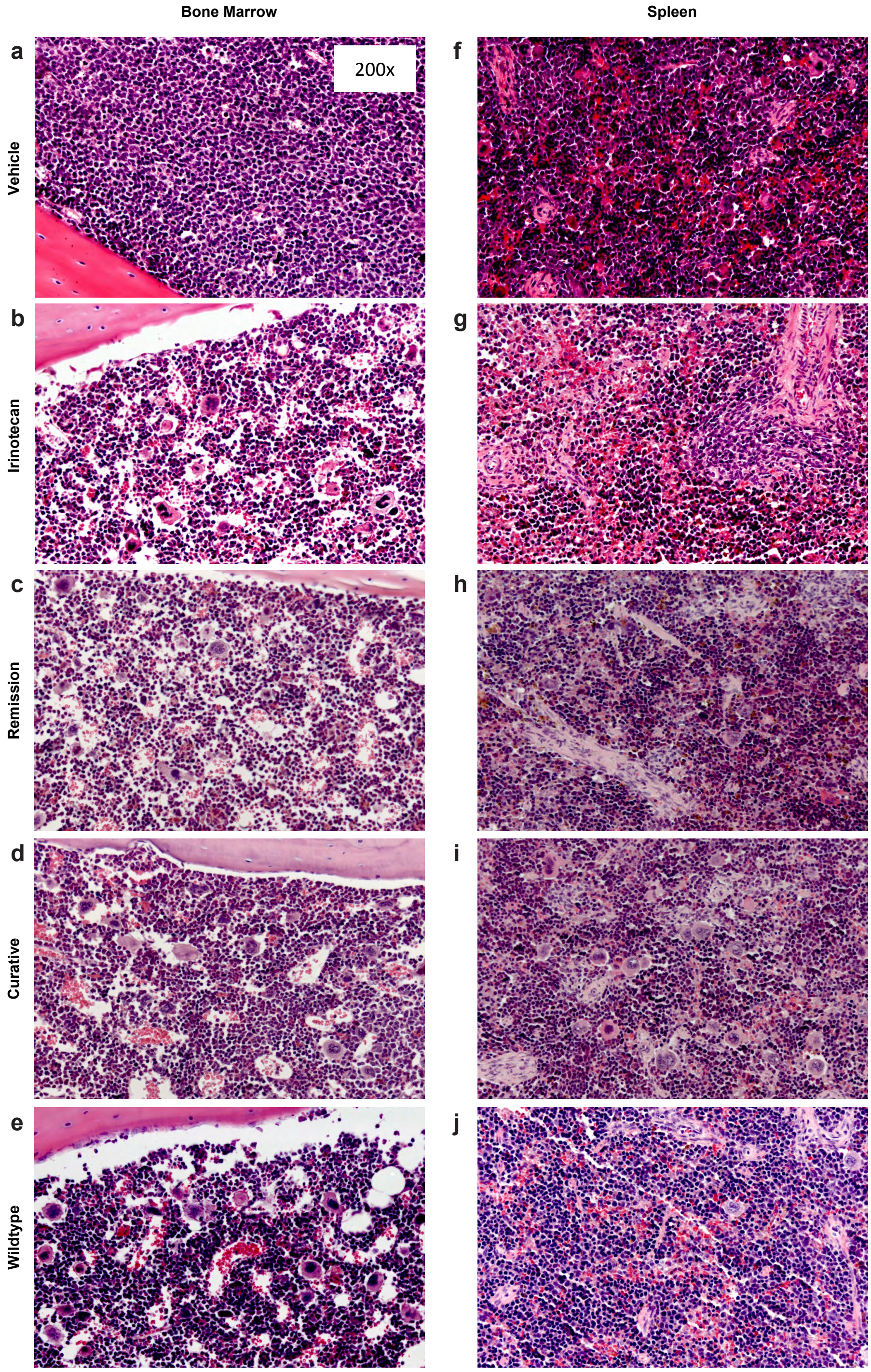

Supplemental Figure 4. Bone marrow and spleen tissue-sections confirm irinotecan effectively inhibits *MLL*-rearranged leukemia tissue-infiltration *in vivo*. (a-e) Hematoxylin and eosin (H&E) stained bone marrow tissue-sections of vehicle, irinotecan, remission, curative and wildtype mice, respectively. (f-j) H&E stained spleen sections of vehicle, irinotecan, remission, curative and wildtype mice. All images were generated using a magnification of 200x.

Supplemental Figure 5.

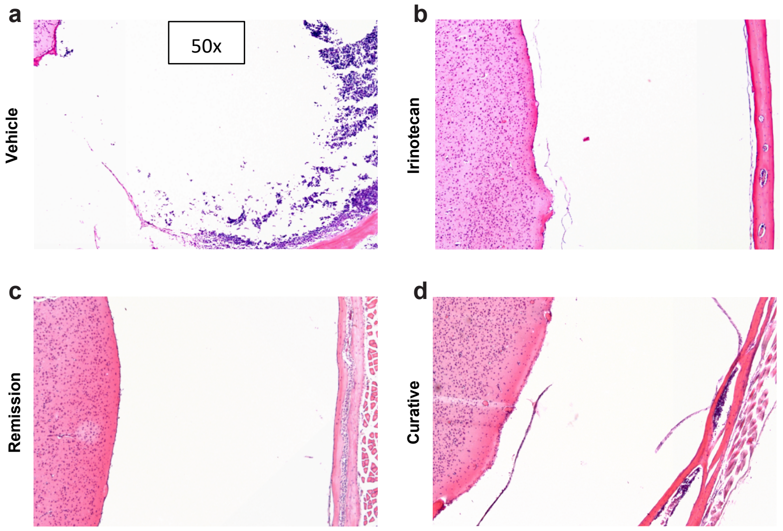

Supplemental Figure 5. a-d) Representative HE stained skull sections of mice from the vehicle, irinotecan, remission and curative treatment groups, respectively.
